# Cardiovascular risk screening: a 10-year prediction cross-sectional study in a Nigerian agrarian community

**DOI:** 10.11604/pamj.2024.47.59.38486

**Published:** 2024-02-11

**Authors:** Sunday Odunke Nduka, Obinna Chris Emeneka, Ifeoma Jovita Nduka, Jude Chinedu Onunkwo

**Affiliations:** 1 Department of Clinical Pharmacy and Pharmacy Management, Nnamdi Azikiwe University, Awka, Anambra State, Nigeria

**Keywords:** Cardiovascular disease, diabetes mellitus, hypercholesterolemia, hypertension, rural community

## Abstract

**Introduction:**

cardiovascular disease (CVD) is a major public health issue with a high global death rate and a significant death contribution from low-and middle-income countries. Modifiable and non-modifiable risk factors assessment and screening are important in their effective prevention and control. This study was designed to screen and assess cardiovascular risk factors in an agrarian community in Nigeria and to predict their 10-year CVD risk.

**Methods:**

this was a cross-sectional study carried out in the Umueri community in Anambra State, Nigeria. Each participant responded to an epidemiologic survey using the World Health Organization (WHO) cardiovascular risk factors assessment tool with point-of-care screening procedures. The risk assessment for 10-year CV risk was conducted using region-specific WHO/ISH charts. Patients´ characteristics were analyzed and presented in frequencies and percentages.

**Results:**

the mean age, systolic blood pressure, fasting plasma glucose, and total cholesterol of the study population were 54 years ± 1.27, 132 mmHg ± 2.088, 130 mg/dl ± 4.608, and 215 mg/dl ± 10.355 respectively. However, 98 (48.8%) have never had their blood pressure checked. About a quarter of the population had a high predicted risk of developing CVD within 10 years.

**Conclusion:**

most of the assessed cardiovascular risk factors in the community are on average above the normal ranges and their probability risk of developing CVD within the next 10 years is high.

## Introduction

Nigeria, just like other low- and middle-income countries (LMICs) is undergoing an epidemiological transition, leading to more attention to non-communicable diseases (NCDs) [1]. Non-communicable diseases are most times chronic and usually occur due to a combination of genetic, physiological, environmental, and behavioral factors, and account for a greater percentage of the world´s mortality rate with about 41 million deaths per year [2]. Although communicable diseases are still the leading cause of death in sub-Saharan Africa, non-communicable diseases have been projected to take the lead by 2030 [3]. The increasing incidence of NCDs and their risk factors mainly in LMICs is attributable to changes in lifestyle and an increase in the aging population [1] among others. Some of the diseases classified under the NCDs category include cardiovascular diseases (CVDs), cancers, chronic respiratory diseases, and diabetes [4,5].

Even though the different diseases classified under the NCDs contribute significantly to the overall mortality rate, CVDs account for about 31% of total death globally with over 75% of this death occurring in low-and middle-income countries [6]. This increases health care costs with a resultant negative impact on the poverty reduction initiatives in these countries [7]. With major contributions from heart attack and stroke, CVDs are among the major global public health concerns with an estimation of about 17.9 million deaths in 2016 [2,8].

With the increasing prevalence of CVD and its associated morbidity and mortality rate in LMICs, there is a need for proper implementation of effective strategies to decrease the mortality rate in sub-Saharan Africa [9]. Risk factors assessment and screening for cardiovascular diseases and NCDs in general are central to their effective prevention and control [10]. The risk factors grouped into modifiable and non-modifiable factors are associated with life-long habits and processes of childhood-onset [11]. Extensive evidence supports the notion that addressing and mitigating modifiable risk factors significantly contribute to a remarkable reduction in CVDs [12]. Over 90% of the population's risk for CVDs is associated with these modifiable risk factors which include smoking, hypertension, diabetes, abdominal obesity, inadequate consumption of fruits and vegetables, insufficient physical activity, and stress [7,8,12,13]. Identification and screening of these risk factors through effective integrated health promotion programs and policies is critical in addressing and curbing this disease burden. Meanwhile, both individual- and community-based approaches have been proposed and used [14]. The high incidence of cardiovascular disease (CVD) in sub-Saharan Africa, along with the documented elevated mortality rate in low- and middle-income countries (LMICs), has been linked to inadequate prevention, detection, and treatment strategies for the associated risk factors. A notable contributing factor is the absence of an effective preventive strategy. The WHO recommends a population-wide strategy focused on public health prevention efforts, emphasizing community-level screening and health education as highly effective measures to prevent CVD events [14,15]. Developed nations widely adopted this screening and awareness approach. Few studies focused on community-based CV risk screening, emphasizing the detection of CVD in individuals without self-reported traditional risk factors [12,16]. However, in Nigeria, such studies are scarce and CVD risk screening in Nigeria has been described to be sub-optimal [17] and may have contributed partly to the low life expectancy and quality of life of Nigerians [18]. The objective of this study was therefore to assess the outcome of cardiovascular risk assessment in an agrarian community in Anambra State, Nigeria using a cardiovascular risk assessment model and then, to predict their 10-year CVD risk.

## Methods

**Study design:** the study was a cross-sectional study conducted among adult members (≥40 years of age) in a rural community in Nigeria on Saturday 7^th^ December, 2019. They were invited for a medical mission (in a fasted state) through an open van campaign, letters to various churches and other organizations, and town hall meetings to a designated place for examination by a group of volunteer medical personnel.

**Study area and setting:** the study was conducted in Umueri community in Anambra State, Nigeria. Umueri is a community in Anambra East Local Government Area of Anambra State and is one of the major ancient towns in the state. The local government with about six communities has a population size of about 201,300 (adjusting for the population projection of 2016) [19]. Umueri community is located within the Anambra Valley, bordered by the Anambra River (Omabala River) and Anam community in the north, Nteje to the south, Aguleri and Nando in the east and Nsugbe in the western flank. Umueri is predominantly occupied by the Igbo ethnic group and they are mostly farmers, fishermen, craftsmen, and traders.

**Eligibility criteria:** only participants who were ≥40 years of age and also admitted not to have taken any food that morning (fasted state) were included in the study while critically ill participants were excluded.

**Data collection procedures and measurement:** presenting participants were addressed by professionals and the procedures were clearly defined, followed by requests for consent individually. Upon obtaining oral consent from a total of 244 adults who presented for the screening, each participant responded to an epidemiologic survey administered by an interviewer. Point-of-care screening procedures, based on the cardiovascular risk factors assessment form (Annex 1) adapted from the WHO STEP-wise approach were used. This helped gather information on demographics, non-modifiable risk factors, and modifiable risk factors including behavioral characteristics like tobacco and alcohol use, diet, level of physical activity, and history of diseases. Additionally, anthropometric measurements of the participants were taken. All measurements were compared to the standards and normal values specified in the various treatment guidelines [11,20-23].

Participants´ blood pressures were measured using a manual sphygmomanometer (Accoson, USA) and a stethoscope using the left arm placed at the heart´s level. Those with raised blood pressure underwent two confirmatory measurements at least five minutes apart and an average of the three measurements was used to establish a diagnosis of hypertension. Similarly, fasting plasma glucose was obtained using glucometers (Accu-Check®, USA) which were properly calibrated. Anthropometric measurements including waist circumference, body mass index (BMI), and waist-hip-ratio (WHR) were achieved using the guidelines described in WHO training manual [11]. Fasting cholesterol (TC, HDL-C, LDL-C, TG) was determined using CardioChek® PA Analyzer (PTS Diagnostics, USA) following the steps described in the user manual. Respondents on hypertensive medications, dyslipidemic medications, and diabetic medications were considered to be suffering from those conditions.

**Study staff/volunteer team training:** the team for the medical mission consisted of lay community volunteers, physicians and allied health care professionals (nurses, pharmacists, medical students). These volunteers attended a one-day training session where they were trained to assess CVD risks, and provide advice with education and modalities for referral according to their various professions using the WHO training manual (10). They were also provided with information on CVDs and major risk factors (unhealthy eating, physical inactivity, smoking, excess alcohol consumption, hypertension, hyperlipidemia, and diabetes) as well as training on blood pressure, plasma glucose, anthropometric and blood lipid measurements as provided [11].

**Sample size:** with a population size of about 201,300 (adjusting for the population projection of 2016) [19], in the community, a total of 32,208 people should qualify for the screening exercise in the area (assuming 16% of the total population to be ≥40 years). Using this number and assuming a confidence level of 95% with a confidence interval of ± 5, a total of 380 adults greater than 40 years of age were deemed appropriate for the study.

**Data analysis:** data were collected, coded, and entered into Excel spreadsheet. Statistical analyses were conducted using SPSS version 22. Descriptive statistics were computed for the demographic variables. Quantitative data were analyzed by computing frequency tables, means, proportions, and percentages. Results were expressed as mean ± SEM and percentages. The 10-year risk of primary onset of CVD for each patient was performed using the region-specific WHO/ISH risk assessment chart (AFR D) using age, sex, blood pressure, smoking status, total cholesterol and diabetes mellitus as specified [24] with CV risks classified as low (<10%), moderate (10% to 20%) and high (≥20%) risks.

**Ethical issues:** ethical approval with Ref number; COOUTH/CMAC/ETH.C/Vol./FN/04/0052 dated 06/04/2018 was obtained from the Ethics Committee of Chukwuemeka Odumegwu Ojukwu University Teaching Hospital, Anambra State, Nigeria, and oral consent obtained from participants before administering the questionnaire.

## Results

Out of the 244 adults who presented for the medical health mission, only 201 persons were successfully captured and their questionnaires were completely filled with the required information. The respondents´ demographic characteristics and CVD modifiable risk factors are presented in Table 1. The majority of the respondents were males (61.7%) with a mean population age of 54 years ± 1.27 and a modal age of 68 years. Majority of the respondents were farmers and traders (74.6%) with just 8.0% being civil servants. The modifiable risk factors to cardiovascular diseases among the respondents showed that the majority (85.6%) of the respondents had never smoked cigarettes/tobacco. Just about a third (35.8%) of them admitted to taking alcohol with 43.8% not being able to describe how often they drank. A good number (66.5% and 47.7% respectively) did not always take fruits and vegetables daily. In addition, 48.8% stated that they had never checked their blood pressure.

**Table 1:** participant’s demographic characteristics and modifiable risk factors

Variable	Frequency (%)
**Demographic characteristics**	
**Age (year)**	
40-50	39 (19.4)
51-60	34 (16.9)
61-70	69 (34.3)
71-79	59 (29.4)
**Civil status**	
Farmer	111 (55.2)
Trader	39 (19.4)
Civil servant	16 (8.0)
Others	35 (17.4)
**Modifiable risk factors**	
**Cigarette/tobacco smoking**	
Never smoked	172 (85.6)
Passive smoker	7 (3.5)
Current smoker	10 (5.0)
Smoked and stopped	12 (6.0)
**Alcohol drinking**	
Never	129 (64.2)
Current drinker	38 (18.9)
Drank and stopped	34 (16.9)
**Drinking frequency**	
Daily	11 (5.5)
1-2/week	22 (10.9)
3-4/week	30 (14.9)
1-2/month	50 (24.9)
Don’t know	88 (43.8)
**Frequency of taking high fat/high salt food**	
Always	9 (4.5)
Usually	33 (16.4)
Often	48 (23.9)
Sometimes	80 (39.8)
Never	31 (15.4)
**Physical activity**	
Sedentary	34 (16.9)
Active	167 (83.1)
**Fruit intake per day**	
Always	29 (14.5)
Usually	38 (19.0)
Often	41 (20.5)
Sometimes	56 (28.0)
Never	36 (18.0)
**Vegetables intake per day**	
Always	53 (26.4)
Usually	52 (25.9)
Often	66 (32.8)
Sometimes	30 (14.9)
**Blood pressure monitoring frequency**	
Never	98 (48.8)
Per day	37 (18.4)
Per week	6 (3.0)
Per month	60 (29.9)

Fewer respondents indicated the existence of non-modifiable risk factors (Table 2) in their lives with about one-third (36.3%) of the screened population having a family history of hypertension, while 14.9% indicated having a history of cardiovascular disease and only 1.5%, history of diabetes mellitus. The clinical features of the participants showed that majority of the screened population indicated not having been diagnosed of hypertension (61.2%) and diabetes (71.1%) with none of them receiving a cholesterol medication or having been placed on a low-cholesterol diet by a physician.

**Table 2:** non-modifiable risk factors, clinical and biometric characteristics of the participants

Variable	Proportion of Respondents, n (%)	
**Non-modifiable risks**	**Yes**	**No**
Family history of hypertension	73 (36.3)	128 (63.7)
Family history of cardiovascular diseases	30 (14.9)	171 (85.1)
Family history of diabetes mellitus	34 (16.9)	167 (83.1)
Family history of hypercholesterolemia	3 (1.5)	198 (98.5)
**Clinical characteristics**	**Yes**	**No**
Feeling stressed	54 (26.9)	147 (73.1)
Previous diagnosis of diabetes mellitus	58 (28.9)	143 (71.1)
Previous diagnosis of hypertension	72 (35.8)	123 (61.2)
Previously informed of the presence of high cholesterol	8 (4.0)	193 (96.0)
Previous placement on a low-cholesterol/low-fat diet	0 (0)	201 (100)
Previously placed on high cholesterol medication	0 (0)	201 (100)
**Systolic blood pressure (mmHg)**	**Frequency (%)**	
Normal (<120)	51 (25.2)	
Prehypertension (120-139)	80 (39.7)	
Stage 1 (140-159)	43 (21.4)	
Stage 2 (≥160)	28 (13.7)	
**Mean systolic blood pressure (mmHg) = 132±2.088**		
Diastolic blood pressure (mmHg)		
Normal (<80)	97 (48.1)	
Prehypertension (80-89)	51 (25.2)	
Stage 1 (90-99)	32 (16.0)	
Stage 2 (≥100)	21 (10.7)	
**Mean diastolic blood pressure (mmHg) = 79±1.312**		
Fasting Plasma Glucose (mg/dl)		
Normal (<100)	45 (22.3)	
Prediabetes (100-125)	76 (37.9)	
Diabetes (>125)	80 (39.8)	
**Mean fasting plasma glucose (mg/dl) = 130±4.608**		
Total cholesterol (mg/dl)		
Desirable (<200)	90 (44.6)	
Borderline (200-239)	32 (16.1)	
High (≥240)	79 (39.3)	
**Mean total cholesterol (mg/dl) = 215±10.355**		
High-density lipoprotein (mg/dl)		
Low (<40)	11 (5.45)	
High (≥40)	190 (94.6)	
**Mean High-density lipoprotein (mg/dl) = 86±10.699**		
**Low-density lipoprotein (mg/dl)**		
Normal (50-100)	77 (38.5)	
High (>100)	124 (61.5)	
**Mean low-density lipoprotein (mg/dl) = 116±6.468**		
**Body mass index (BMI)**		
<18.5 kg/m^2^	0 (0)	
18.5-24.9 kg/m^2^	131 (65.2)	
> 25 kg/m^2^	70 (34.8)	
Waist-hip ratio		
< 1	199 (99.0)	
>1	2 (1.0)	

Almost a quarter (25.2%) of the participants had their systolic blood pressure within the normal range of less than 120 mmHg while 39.7% and 13.7% of them were at prehypertension and stage 2 hypertension category respectively with a mean value of 132 mmHg ± 2.088. Conversely, while almost a half of the respondents had their diastolic blood pressures within the normal range, 21 accounting for 10.7% of the participants had their diastolic blood pressure (DBP) ≥100 mmHg with a mean value of 79 mmHg ± 1.312. Furthermore, with a mean fasting plasma glucose of 130 mg/dl ± 4.608, 80 (39.8%) of the participants had their fasting plasma glucose above 125 mg/dl which is a diabetic state. Similarly, 39.3% of the participants had their total cholesterol greater than or equal to 240 mg/dl with a mean of 215 mg/dl. While only 5.45% of them had their high-density lipoprotein below 40 mg/dl, 61.5% had their low-density lipoprotein above the normal range with a majority (65.2%) having their body mass index between the normal range of 18.5 - 24.9 kg/m^2^.

**Figure 1:**
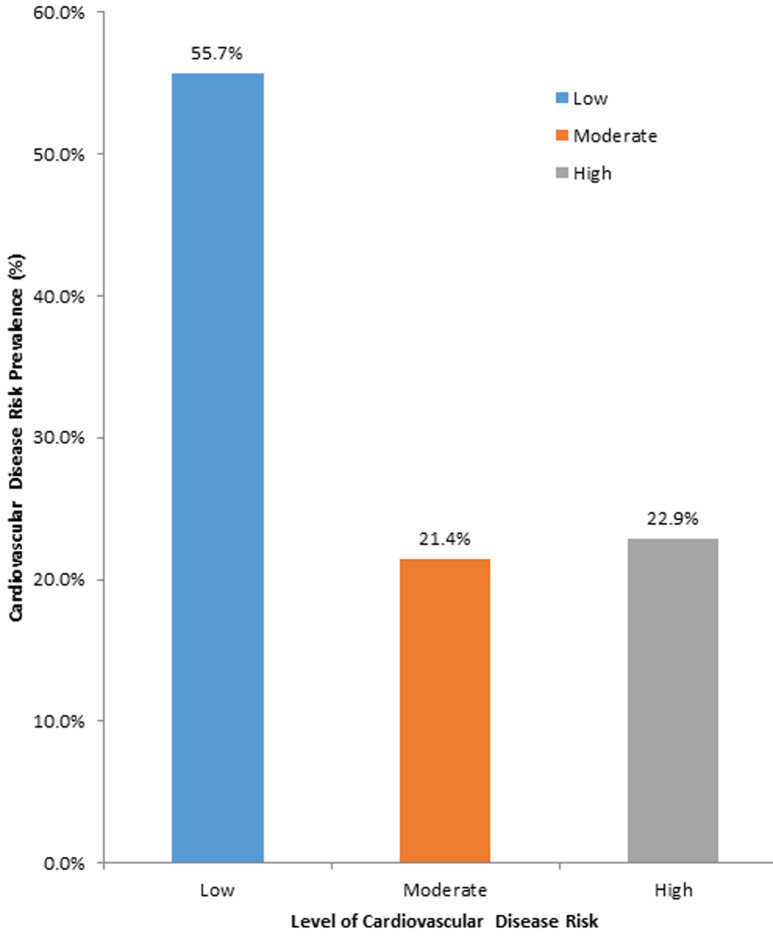
ten (10)-year cardiovascular disease (CVD) prediction

On the 10-year CVD risk prediction, close to a quarter (22.9%) of the respondents had high CVD risk with an almost similar percentage (21.4%) classified under the moderate category (Figure 1).

## Discussion

This study assessed the cardiovascular risk factors among adults greater than 40 years of age in an agrarian community in Anambra State, southeast, Nigeria, and then, predicted a 10-year cardiovascular risk of this population using WHO/ISH prediction charts. Although some of the respondents´ cardiovascular risk factors were within normal range or limits, their systolic blood pressure, total cholesterol, and plasma glucose levels were seen to be relatively high with about a fifth of the population showing a high risk of developing cardiovascular diseases over a 10-year period.

Identification and screening for risk factors of cardiovascular diseases through community-based approaches have been proposed as effective and acceptable ways of curbing the menace of these diseases in the very near future [10]. History taking and simple measurements of the modifiable and non-modifiable risk factors including heredity/family history, tobacco/alcohol use, plasma glucose, blood pressure, and cholesterol measurements are the basic ways of assessing these risks.

This present study revealed that the majority of the surveyed population had their systolic and diastolic blood pressure above the normal range with a mean systolic blood pressure of 132 mmHg which is a prehypertension stage. Elevated blood pressure is one of the most important risk factors for CVD [25,26] and systolic blood pressure has been identified as a stronger predictor of CVD risk compared to diastolic blood pressure [27]. Hence, the good number of the participants having their systolic blood pressure at stages 1 and 2 categories in addition to more than one-third at the prehypertension stage gives concern. Meanwhile, prehypertension has been reported to be associated with increased risks of major cardiovascular events [28] and the prevalence of prehypertension has been reported to be high in some African countries and among the Indians [29]. Although, non-pharmacologic approaches including the use of dietary modification, reduction in salt intake, weight loss, reduction in alcohol intake, and regular physical activity among others are essential in reducing progression from this prehypertension class to full-blown hypertension [29], achieving such lifestyle changes and dietary modification in typical rural settings may be difficult. Meanwhile, the high prevalence of CVD in rural communities has been strongly associated with these modifiable risk factors and less healthy lifestyle behaviors [30]. In agreement to that finding, this study observed poor indices in some of these modifiable risks among the surveyed population highlighting the risk of CVD in this community. Indulging in acts such as alcohol intake and cigarette/tobacco smoking may be predisposing this community to hypertension thereby increasing the risk of CVD [14].

Another important approach in preventing hypertension and CVD risk is through routine blood pressure measurement and monitoring which should be accurate and regularly assessed [31] and lack of this may be associated with more cardiovascular events in a given population. Our study identified that almost half of the surveyed population had never monitored their blood pressure placing them at an increased risk of CV event. This non-monitoring of blood pressure has been blamed on poor access to routine education programmes in most rural areas in developing countries [32].

A number of the surveyed population had their fasting plasma glucose above the normal range with less than a quarter below 100 mg/dl and the average fasting plasma glucose above the normal range of 70 to 100 mg/dl. Like high blood pressure, high plasma glucose has been shown to be associated with a high risk of cardiovascular diseases [33]. Meanwhile, duration of the disease, presence of albuminuria or impaired renal function, and poor glycemic control have also been shown to be independent CVD risk factors in diabetic patients [33]. Although this study did not look at impaired renal function, an average plasma glucose level above the normal range in the fasting state in this population is indicative of possible diabetic condition in the surveyed population and a high plasma glucose value in the fasting state in many of the participants may be indicative of poor glycemic control which also increases their CVD risk [34].

An increase in weight and obesity has been shown to result in increase in diabetic conditions, especially, type 2 diabetes, with an expectant increased risk of cardiovascular diseases [35]. These conditions also increase metabolic syndrome which further exacerbates the cardiovascular risk [36]. Although the waist-to-hip ratio of the surveyed population was appropriate, a good proportion of them were overweight with BMI greater that 25 kg/m^2^, and a high total cholesterol was also seen in some of the population.

Cardiovascular risk prediction uses biological characteristics (age, gender) and modifiable risk factors (diabetes, systolic blood pressure, lipid levels, and lifestyle conditions in particular, smoking) to assess and predict CVD risk [37]. It is advisable for patients aged 40 to 79 years without established CVD or diabetes to undergo periodic CVD risk assessment every four to six years, as periodic risk assessment offers the opportunity to identify CVD risk factors and provide guidance on the appropriate management of specific risk factors including dietary modifications for hypertension or dyslipidemia among others and overall CVD risk like maintaining a healthy diet and regular exercise [38]. From our study, the overall 10-year CVD risk was high among the community. This is similar to the findings from previous studies which found a high risk of developing CVDs within 10 years in their respective surveyed populations [39,40] but, with differing prevalence.

Hypertension and diabetes are two main contributors to CVDs [41] with the two closely linked together. Similarly, raised total cholesterol has also been identified as a strong risk factor for CVD [42]. Prevalence of undiagnosed hypertension and diabetes including cholesterol in most societies has been reported to be high and this increases the risks of cardiovascular disease development among the individuals. This is a result of the asymptomatic nature of CVDs and most of its risk factors [13]. However, early detection and management of these conditions reduce this risk which illustrates the importance of this study.

There are some limitations in this study. First, the convenient use of only 244 persons that presented for the mission which is also lower than the calculated sample size may not allow for generalization of the study findings. However, this study may serve as a pilot for a more robust study in agrarian communities that will help improve their cardiovascular health indices. The study relied on self-reported data from the respondents in some of the questionnaire items without verification and hence, the respondents may have supplied responses as they desired introducing social desirability bias. Again, although the participants were informed to present for the mission in a fasted state, some of them may have taken something before presenting for the screening. This might have affected the results of some of the tests. Nevertheless, the results resonate with the importance of CVD risk assessment in remote villages in developing countries. This study could not progress to assess the feasibility of linkage to care and retention in care of the surveyed population which would have made this study complete with total care provision for the participants. This, however, in addition to an assessment of the impact of educational intervention provision forms the basis of future studies.

## Conclusion

Poor cardiovascular risk indices including poor blood pressure, plasma glucose, and total cholesterol with some lifestyle situations resulted in an increased risk of developing CVDs in the next 10 years in Umueri community in Anambra State, Nigeria. This community, therefore, requires enlightenment/education on behavioral modification and CVD risk reduction strategies, with emphasis on the importance of blood pressure monitoring on lowering CVD risks.

### What is known about this topic

- *Cardiovascular disease contributes significantly to the overall global morbidity and mortality rate with low-and middle-income countries contributing meaningfully to this number;*
- *Although, risk factors assessment and screening through health promotions have been identified as the most effective strategy for the prevention and control of this disease and adopted in most developing countries, adoption and practice of this concept in developing countries including Nigeria have been sub-optimal*.

### What this study adds

- *This study identified asymptomatic individuals with a high 10-year CVD risk requiring preventive medications to reduce future CVD morbidity and mortality in the community;*
- *This underscores the need for the assessment and screening of cardiovascular risks in rural asymptomatic patients and adopting these strategies in individual rural settings will help improve the acceptability and practice of this concept*.

## Annex

**Annex 1**: cardiovascular risk factor assessment form
